# Spontaneous Splenic Rupture in a Young Patient: A Comprehensive Case Report and Literature Review

**DOI:** 10.7759/cureus.60105

**Published:** 2024-05-11

**Authors:** Eman M Alyaseen, Nawaf A Hantol, Joud A Alyousef, Bader S Alanazi, Radhwan Alghamdi

**Affiliations:** 1 College of Medicine and Medical Sciences, Arabian Gulf University, Manama, BHR; 2 College of Medicine, Imam Abdulrahman Bin Faisal University, Dammam, SAU; 3 General Surgery, Dammam Medical Complex, Dammam, SAU

**Keywords:** case report, splenic rupture, non-traumatic, young, acute abdomen, spontaneous

## Abstract

Spontaneous splenic rupture (SSR), a rare but potentially life-threatening condition, typically occurs in the absence of trauma or underlying splenic disease. This report aims to contribute to the limited body of knowledge regarding its occurrence, diagnosis, and management in this demographic. We describe the case of a 20-year-old patient with no significant medical history who presented with acute abdominal pain and hypovolemic shock. Imaging revealed an unexpected splenic rupture without any preceding trauma or identifiable risk factors. The patient’s clinical progression, diagnostic challenges, and therapeutic approach are discussed in detail. This case underscores the importance of considering SSR in the differential diagnosis of acute abdomen in young patients, even in the absence of predisposing factors. We review the literature to highlight the epidemiology, possible etiologies, diagnostic modalities, and treatment options for SSR. The peculiarities of managing such cases in young patients are also discussed, emphasizing a tailored approach to balance the risks of conservative management against surgical intervention. In conclusion, SSR, though rare in young patients, should be a diagnostic consideration in cases of unexplained acute abdomen. Early recognition and appropriate management are crucial for favorable outcomes. This case adds to the existing literature by providing insight into the presentation and management of this condition in a young, healthy individual, thereby aiding in enhancing clinical vigilance and patient care.

## Introduction

In the 19th century, Rokitansky documented the first case of atraumatic splenic rupture in a leukemic patient [1]. Meanwhile, Knoblish distinguished “non-traumatic rupture of a pathological spleen” from the extremely rare “non-traumatic splenic rupture of unknown etiology,” which was also known as true “sponsplenic rupture” in 1966 [1,2]. The fact that spontaneous splenic rupture (SSR) occurs without prior trauma or obvious splenic disease makes it an uncommon and frequently confusing medical emergency. Despite having a low prevalence of less than 1% of cases of splenic rupture, its clinical significance comes from the difficulties in diagnosing it and the possibility of serious consequences [3]. Delayed diagnosing of ASR may result in significant mortality, as the ASR-related mortality rate was estimated to be 12% in a systematic review [4]. In this case report, we explore a rare instance of SSR in a young patient, highlighting the need to take this diagnosis into account when a patient in this demographic presents with unexplained severe abdominal discomfort. The purpose of the report is to further knowledge of the clinical presentation of SSR in young patients without predisposing circumstances and the difficulties in diagnosing the condition.

## Case presentation

A 20-year-old male with no significant past medical history presented to the emergency department with a six-hour history of sudden onset, severe left upper quadrant abdominal pain. The pain was sharp, non-radiating, eight out of 10 on a pain scale, and had progressively worsened during the last hours. The patient denied any recent trauma, rigorous physical activity, or participation in contact sports. There was no history of alcohol abuse, illicit drug use, or medication intake that could predispose to splenic pathology. The patient also reported no recent infections or symptoms suggestive of an infectious process. On examination, the patient appeared pale and in distress. Vital signs revealed tachycardia with a heart rate of 110 beats per minute and hypotension with a blood pressure of 90/60 mmHg. Abdominal examination showed tenderness and guarding in the left upper quadrant with no palpable mass. The bowel sounds were normal. There was no evidence of external injury or bruising. Initial laboratory investigations showed hemoglobin of 9.8 g/dL, down from a previous level of 14.2 g/dL noted in a routine check-up six months prior. The white blood cell count and platelet count were within normal ranges. Liver function tests and coagulation profiles were also normal, as shown in Table 1.

**Table 1:** Laboratory values shown are within the normal range except for decreased hemoglobin and hematocrit levels, suggesting acute blood loss from the ruptured spleen

Test	Result	Unit	Interpretation	Reference range
WBC	3.79	×10^9^/L	Normal	3.6-9.6
RBC	4.15	×10^12^/L	Low	4.35-5.6
Hemoglobin	9.8	g/dL	Low	13-14
HCT	29.5	%	Low	33-45
MCV	71.1	fL	Low	80-100
Platelets count	174	×10^9^/L	Normal	150-450
PT	12.001	Sec	Normal	11-13.5
INR	1.059	Sec	Normal	<1.1
PTT	30.647	Sec	Normal	25-35
Protein	78	g/L	Normal	64-82
Globulin	29	g/L	Normal	20-35
Albumin	49	g/L	Normal	34-54
Total bilirubin	17	umol/L	Normal	5-21
Alkaline phosphatase	123	U/L	Normal	50-136
Alanine aminotransferase	13	U/L	Normal	<41
G-Glutamyl transferase	<6	U/L	Normal	<35

**Figure 1:**
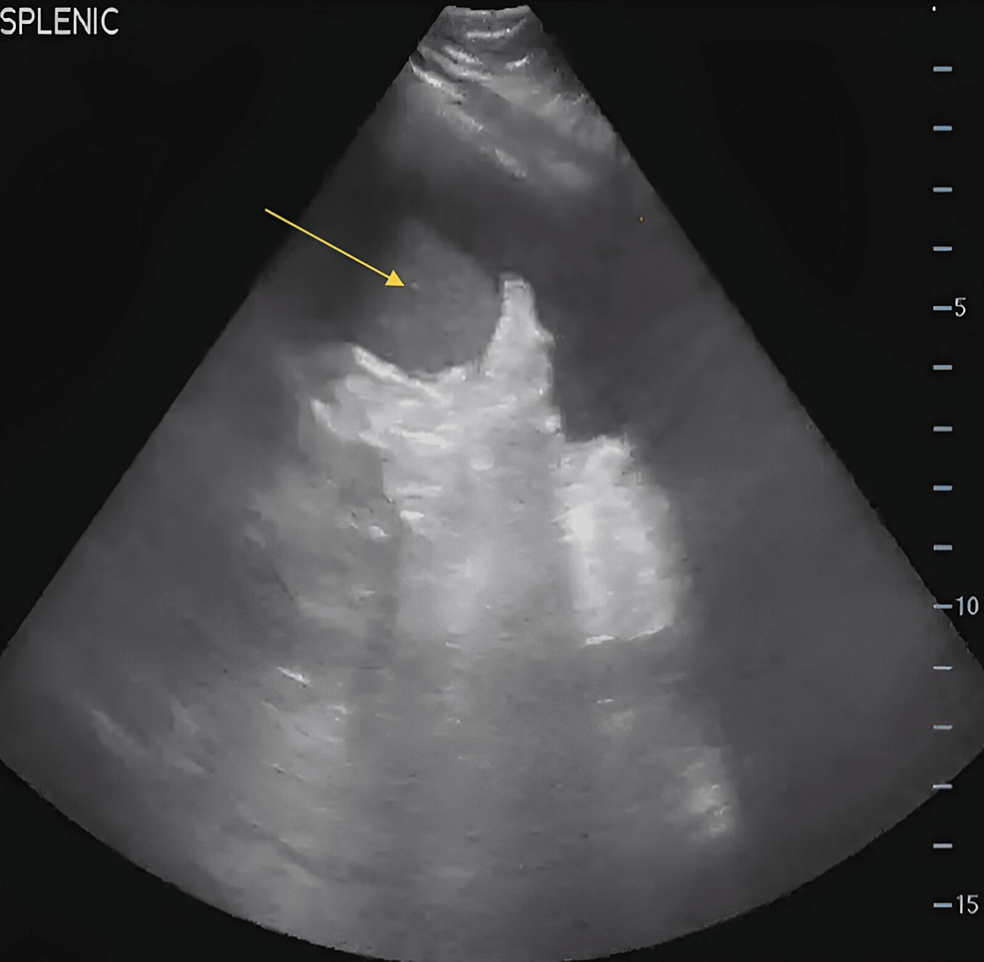
Abdominal ultrasound showing a hypoechoic lesion indicating subcapsular splenic hematoma/bleeding (yellow arrow)

**Figure 2:**
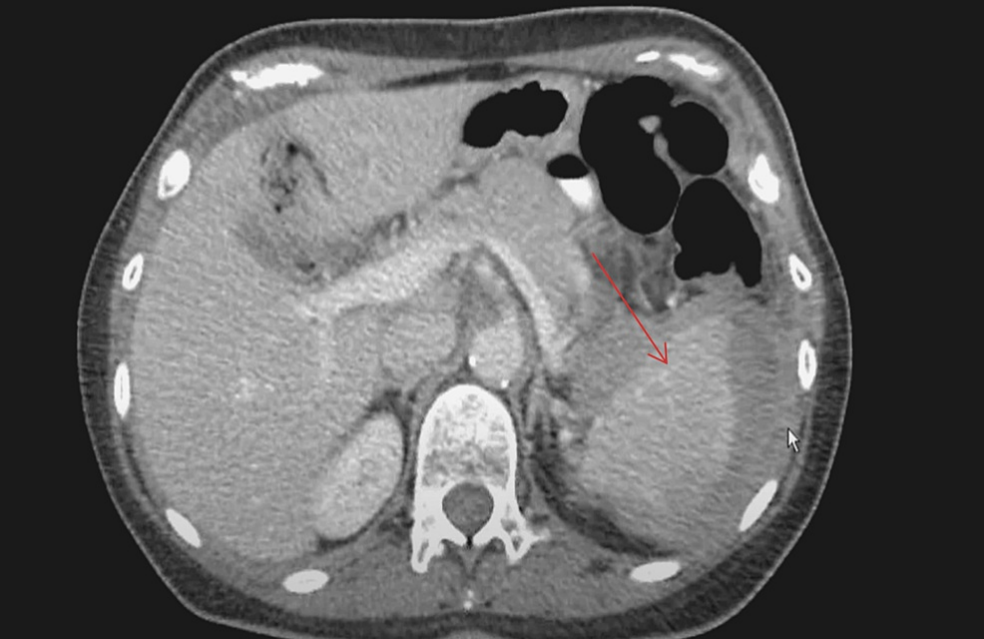
Abdominal CT scan with an IV contrast axial cut showing an irregular contour of the spleen, heterogenous enhancement, splenic parenchyma low-attenuation, and laceration with contrast extravasation (red arrow)

**Figure 3:**
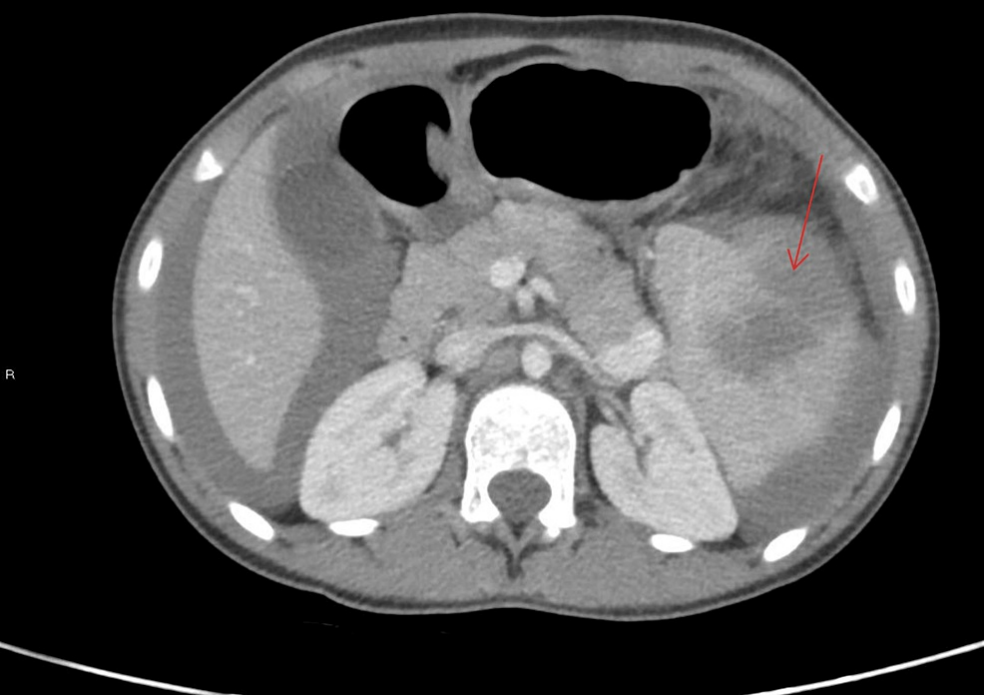
Abdominal CT scan with evidence of splenic rupture and intra-abdominal bleeding (red arrow)

A rapid infectious screen, including rapid antigen tests to directly detect specific antigens of mononucleosis and malaria, was negative. Immediate intravenous fluid resuscitation was initiated due to the patient’s hemodynamic instability. Following initial resuscitation, there was a temporary improvement in the patient’s vital signs, with a heart rate decreasing to 95 beats per minute and blood pressure rising to 110/70 mmHg. With the patient stabilized, an urgent abdominal ultrasound was performed, which revealed free fluid in the abdominal cavity. This finding prompted a contrast-enhanced computed tomography (CT) scan of the abdomen and pelvis, confirming a large hemoperitoneum and a ruptured spleen with evidence of hypoechoic lesion caused by subcapsular splenic hematoma (Figures 1-3).

However, shortly after the imaging studies, the patient’s condition deteriorated, indicating a transient response to the initial resuscitation. He developed worsening tachycardia and a drop in blood pressure, suggesting ongoing internal hemorrhage. Given the patient’s deteriorating hemodynamic status post-imaging, an emergency laparotomy was deemed necessary. During the surgery, a significant amount of hemoperitoneum was encountered, and the spleen was found to be ruptured without any obvious pathological lesions. A splenectomy was performed. The postoperative course was uneventful, and the patient recovered well. The patient was discharged with instructions on wound care, and he was also educated on the signs of infection, the importance of seeking prompt medical attention, and the importance of follow-up vaccinations. He was scheduled for a follow-up visit in the clinic two weeks post-discharge. During the two-week post-discharge clinic visit, the patient was administered vaccinations against encapsulated organisms (pneumococcus, meningococcus, and *Haemophilus influenzae*) to mitigate the increased risk of infections following splenectomy. The patient reported no complications and was healing well. His wound was healing without signs of infection, and he reported no new symptoms. Histopathological examination of the spleen confirmed the absence of any underlying pathological condition. Further follow-up visits were scheduled to monitor his recovery and long-term health without a spleen.

## Discussion

SSR is a rare clinical entity that often presents diagnostic and therapeutic challenges, especially in young patients without predisposing factors [5]. In our case, reporting with SSR without preceding trauma or identifiable risk factors offers a unique opportunity to explore this unusual condition. The rarity of SSR in the absence of trauma or known splenic disease cannot be overstated. Most cases of splenic rupture are associated with trauma, and when spontaneous, they are typically linked to underlying pathological conditions like infectious diseases, including infectious mononucleosis and malaria (27%), inflammatory causes as in hepatitis and pancreatitis (20%), or neoplasms as diffuse large B cell lymphoma (30%). In some cases, SSR can be treatment-related (9%), including the use of anticoagulants and antiplatelet agents, especially in patients hospitalized for cardiac reasons. Our case is particularly intriguing as the patient lacked these usual risk factors [6-9]. This rarity underscores the importance of maintaining a broad differential diagnosis when evaluating young patients with acute abdominal pain. The pathophysiology behind the spontaneous rupture of a structurally normal spleen remains speculative [10-14]. Increased intrasplenic tension due to congestion, vascular anomalies, or enzymatic digestion of the splenic capsule are proposed mechanisms. However, these theories are challenging to validate, especially in cases like ours where the spleen appeared normal on histopathological examination. The clinical presentation of SSR can be misleading. Patients often present with acute onset of abdominal pain, which is non-specific and can mimic other more common causes, such as ectopic pregnancy [15]. Our patient’s presentation with acute left upper quadrant pain and hemodynamic instability exemplifies the typical but non-specific clinical picture of SSR [13]. This highlights the critical role of imaging studies in the diagnosis. The ultrasound and CT scan helped in identifying the hemoperitoneum and splenic rupture. Management of SSR primarily depends on the patient’s hemodynamic stability, and early recognition is crucial for survival. While conservative management may be suitable for hemodynamically stable patients, surgical intervention is warranted in cases of ongoing hemorrhage or hemodynamic instability, as was necessary in our case. The decision to perform a splenectomy, as opposed to spleen-preserving procedures, is often guided by the intraoperative findings and the patient’s overall condition. Emergency splenectomy remains the cornerstone treatment for splenic rupture, as delaying treatment can lead to catastrophic clinical outcomes [16,17]. The post-operative management of patients who have undergone splenectomy, especially regarding vaccination against encapsulated organisms, is crucial. Our approach of vaccinating the patient two weeks post-discharge aligns with recommendations to reduce the risk of overwhelming post-splenectomy infection (OPSI). This case underscores the importance of post-splenectomy vaccines and patient education about the risks of infection. Adequate patient education about the long-term risk of OPSI and increased physician awareness is vital for managing OPSI appropriately. OPSI can occur in both immunized and non-immunized patients, with a reported incidence of around 4-5% in a study of splenectomized patients with hematological diseases. The time interval from splenectomy to OPSI ranged from 10 days to 12 years [18,19].

## Conclusions

To summarize, our case contributes to the small yet expanding collection of research on SSR in young individuals without underlying conditions. It underscores the necessity for vigilant clinical suspicion and immediate diagnostic assessment in these patients who present with sudden abdominal pain. Additionally, the case underscores the significance of a collaborative approach involving emergency physicians, radiologists, and surgeons to ensure timely diagnosis and treatment. Moreover, it serves as a reminder of the crucial role of post-operative care, specifically in terms of vaccination and patient education after splenectomy.
